# The relationship between TRAF6 and tumors

**DOI:** 10.1186/s12935-020-01517-z

**Published:** 2020-09-03

**Authors:** Jiaoduan Li, Nian Liu, Ling Tang, Bei Yan, Xiang Chen, Jianglin Zhang, Cong Peng

**Affiliations:** 1 Department of Dermatology, Xiangya Hospital, Central South University, Changsha, Hunan China; 2 National Clinical Research Center for Geriatric Disorders, Xiangya Hospital, Changsha, Hunan China; 3 Hunan Key Laboratory of Skin Cancer and Psoriasis, Xiangya Hospital, Changsha, Hunan China; 4 Hunan Engineering Research Center of Skin Health and Disease, Xiangya Hospital, Changsha, Hunan China; 5 Xiangya Clinical Research Center for Cancer Immunotherapy, Central South University, Changsha, Hunan China

**Keywords:** TRAF6, Tumorigenesis, E3 ubiquitin ligase, AP-1, NF-κB

## Abstract

Tumor necrosis factor receptor (TNFR)-related factors (TRAFs) are important linker molecules in the tumor necrosis factor superfamily (TNFSF) and the Toll-like/interleukin-1 receptor (TLR/ILR) superfamily. There are seven members: TRAF1-TRAF7, among those members, tumor necrosis factor receptor-associated factor 6 (TRAF6) is upregulated in various tumors, which has been related to tumorigenesis and development. With the in-depth study of the relationship between TRAF6 and different types of tumors, *TRAF6* has oncogenic characteristics involved in tumorigenesis, tumor development, invasion, and metastasis through various signaling pathways, therefore, targeting TRAF6 has provided a novel strategy for tumor treatment. This review summarizes and analyzes the role of TRAF6 in tumorigenesis and tumor development in combination with the current research on TRAF6 and tumors.

## Background

TRAFs are a class of cytoplasmic adaptor proteins. In mammals, six classical members (TRAF1-TRAF6) and one nonclassical member (TRAF7) are currently known. Classical members are those with a common amino acid segment called a TRAF domain at the carboxy terminus. Nonclassical members do not contain a TRAF homology domain. In 1994, the TRAF family of proteins was discovered as part of the downstream signaling pathway of the tumor necrosis factor superfamily (TNFSF) [1]. Subsequent research showed that the TRAF family of proteins participates in the signaling of the TNFSF and the TLR/ILR receptor superfamily and regulated the activation of signaling pathways such as mitogen-activated protein kinase (MAPK) [2]. In addition, the TRAF family of proteins also participates in cell proliferation, differentiation, survival and apoptosis and engage in immune and inflammatory responses [3]. Among those molecules, TRAF1, TRAF2, TRAF4, TRAF5 and TRAF6 might play carcinogenic roles, whereas TRAF3 acts as a tumor suppressor. TRAF1 and TRAF4 affect on skin and lung carcinoma in mouse model [4–6]. TRAF5 may play a carcinogenic role in colorectal cancer and gastric cancer [7, 8]. TRAF2 promotes tumorigenesis in breast and gastric cancers [9, 10]. However, TRAF3 plays an inhibitory role based on B lymphomas mouse model [11]. TRAF6 has unique receptor binding specificity, which plays an important role in the signaling of the TNF receptor superfamily as well as exerts a specific interaction with members of the IL-1R/TLR superfamily [12]. Studies showed that TRAF6 is overexpressed in various types of tumor, including colon, gastric, breast carcinomas and melanoma [13–15]. TRAF6 facilitates the occurrence and development of tumors by affecting cell apoptosis, proliferation, survival, and invasion [16]. In this review, we summarize the advanced research of the role of TRAF6 in tumorigenesis and tumor development, as well as provide tumor therapeutic strategies targeting TRAF6.

## Basical function of TRAF6

**Fig. 1:**
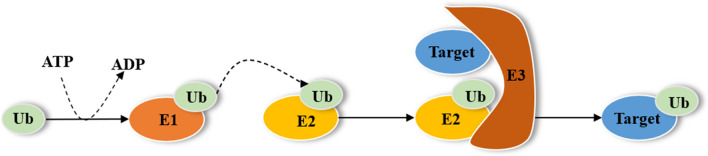
The process of ubiquitination. Protein ubiquitination is a three-step enzymatic reaction. Step one is the reaction of E1 activating ubiquitin, which is ATP-dependent. Step two is the form of E2-Ub thioester by the transfer of activated ubiquitin to E2. Ultimately, E3 facilitates the transfer of ubiquitin from E2 to the target protein

TRAF6 was first discovered as a signal transduction molecule for IL1 and CD40 [17, 18]. The N-terminus of TRAF6 protein contains a RING finger domain and five zinc fingers. The RING domain has E3 ubiquitin ligase (E3) activity, while the zinc finger mainly provides structural support for the activity of the RING domain [19]. There is a TRAF domain at the C-terminus, which consists of a coiled helix and a conserved TRAF-C domain [1]. The TRAF domain enables TRAF6 to play an important biological function by mediating self-binding interactions with the receptors and other signaling proteins’ upstream proteins [20]. With the in-depth study of TRAF6, it was found that it not only helps transmit TNF signals but also acts as an E3, which can generate Lys63-linked polyubiquitin chains together with the Ubc13—uev1a E2 complex, and mediate the degradation of several proteins [21]. Protein ubiquitination is a pivotal post-translational modification, a process in which one or more ubiquitin covalently binds to the lysine residue of the target protein triggered by an enzymatic cascade, and it is a three-step enzymatic reaction performed by three different types of enzymes, including ubiquitin activating enzyme (E1), ubiquitin conjugating enzyme (E2), and E3 [22]. Step one is the reaction of E1 activating ubiquitin, which is ATP-dependent. Step two is the form of E2-Ub thioester by the transfer of activated ubiquitin to E2. Ultimately, E3 facilitates the transfer of ubiquitin from E2 to the target protein (Fig. 1). The binding of K63-linked polyubiquitin chains to target molecules regulates intracellular signal transduction and thus participates in regulating immune function [23].

## TRAF6 mediates signaling transduction pathway

**Fig. 2:**
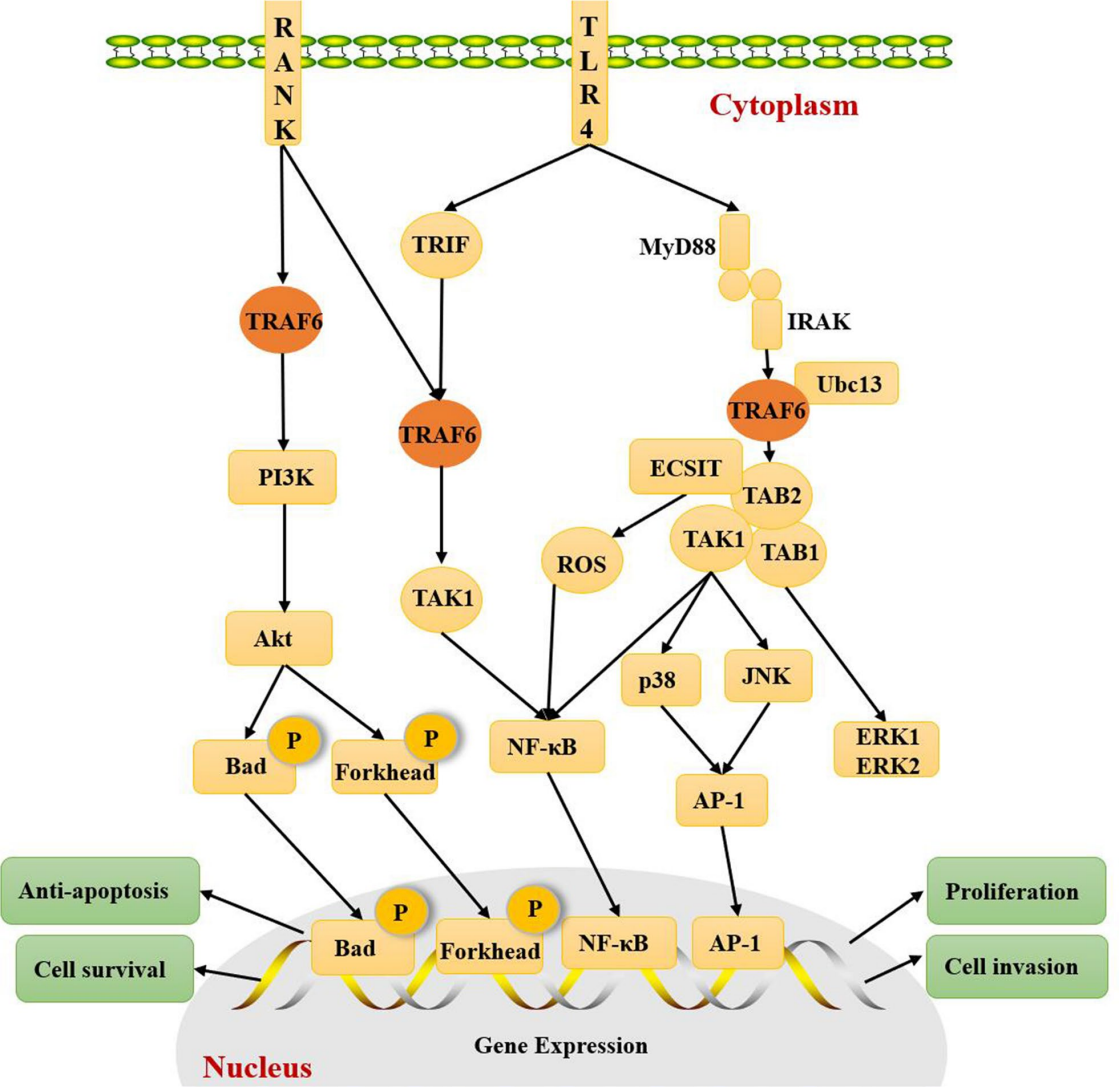
TRAF6 mediates signaling transduction pathway. TRAF6 participates in multiple signaling pathways. In the MyD88-dependent pathway of TLR4 signaling pathway, TRAF6 interacts with IRAK and forms the IRAK-TRAF6-TAK1-TAB 1TAB 2 complex and subsequently activates the MAPKs (ERK1, ERK2, p38, and JNK). Ultimately, NF-κB and AP-1 are activated. In the MyD88-independent pathway, TRIF recruits TRAF6 and activates TAK1 to induce NF-κB activation. In RANK signaling pathway, activated RANK interacts with TRAF6 and thereby mediates activation of PI3K-AKT and NF-κB. Akt can phosphorylate Bcl-xL/Bcl-2 associated death promoter (Bad) and Forkhead family members, thus inhibiting apoptosis. In addition, TRAF6 interacts with ECSIT protein results in increasing levels of ROS

TRAF6 triggers multiple signaling pathways, the most notable of which is the Toll-like receptor 4 (TLR4) signaling pathway, which involves the MyD88-dependent and MyD88-independent pathways. In the MyD88-dependent pathway, MyD88 recruits and activates interleukin-1 receptor-associated kinase (IRAK). Activated IRAK interacts with TRAF6 to form a K63-linked polyubiquitin chain, which activates TAK1 and forms the IRAK-TRAF6-TAK1-TAB 1TAB 2 complex, then causes a series of cascade reactions and results in the activation of MAPKs (ERK1, ERK2, p38, and JNK). In this process, the transcription factors nuclear factor kappa-B (NF-κB) and activator protein-1 (AP-1) are ultimately activated and affect translation [24]. In the MyD88-independent pathway, TRIF recruits TRAF6 and activates TAK1 to induce NF-κB activation [24] (Fig. 2). TRAF6 also involves in RANK singling pathway and evidences showed activated RANK could interact with TRAF6, consequently, TRAF6 induces activation of phosphatidylinositol 3-kinase (PI3K)-AKT and NF-κB [25] (Fig. 2). Akt can phosphorylate Bcl-xL/Bcl-2 associated death promoter (Bad) and Forkhead family members, thus inhibiting apoptosis. In addition, the studies also showed that TRAF6 interacts with the protein evolutionarily conserved signaling intermediate in Toll pathways (ECSIT) to ubiquitinate and enrich its around the mitochondria, resulting in raising cellular reactive oxygen species (ROS) in mitochondrial [26] (Fig. 2).

## TRAF6 regulates tumor related to signaling transduction pathways

### TRAF6 mediates AKT ubiquitination and activation

**Fig. 3:**
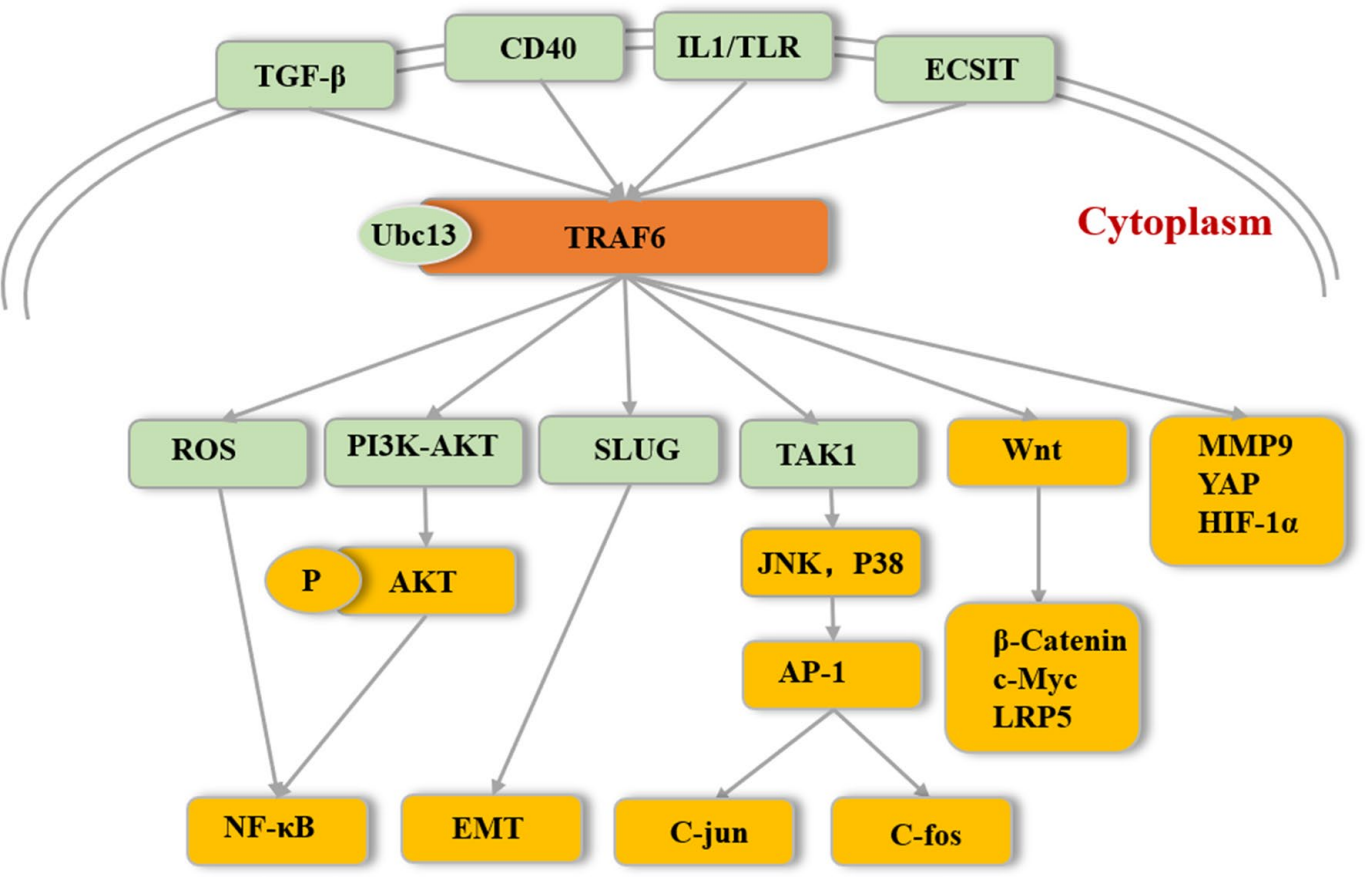
TRAF6 regulates tumor related to signaling transduction pathways. TRAF6 affects multiple Pathways involved in cancer after receiving upstream signals including TGF-β, IL1/TLR and CD40. TRAF6 promotes PI3K-AKT signaling and leads to an increase in AKT ubiquitination and phosphorylation. By interacts with ECSIT, TRAF6 increase ROS levels and thereby indirectly enhances NF-κB activation. TRAF6 also induces JNK and p38 activation in a TAK1-dependent manner, leading to activation of the AP-1 family members c-jun and c-fos. As a key transfer adaptor protein that transduces the Wnt3a/β-Catenin pathway, TRAF6 upregulates mRNA expression of β-Catenin and subsequently activates Wnt target genes such as *c*-*Myc* and *LRP5.* TRAF6 promotes the expression of SLUG, which contributes to EMT. Besides, TRAF6 up regulate MMP9 and HIF-1α. In addition, TRAF6 also regulates YAP signaling

The PI3K-AKT pathway is activated in many malignant human tumors, such as endometrial cancer, melanoma, and glioblastoma [27]. Wang et al. illustrated that TRAF6 promotes PI3K-AKT signaling and leads to an increase in AKT phosphorylation and cell growth [28]. They hypothesized that TRAF6 functions as an adaptor molecule to enhance PI3K activity through ubiquitination [28] (Fig. 3). AKT is activated by phosphorylation of Thr308 and Ser473 and subsequently phosphorylates a variety of downstream protein substrates [29]. Phosphorylated AKT (pAKT) engages in the dysregulation of apoptosis, proliferation, and cell movement [30]. Therefore, pAKT is considered a clinical prognostic indicator and a target of cancer treatment.

Lys48-linked polyubiquitination is the major signal for proteasomal degradation of target proteins, whereas Lys63-linked polyubiquitination has nondegradative regulatory effects, including vesicle trafficking, DNA repair, and signal transduction [31]. A structural study shows that the configuration of the K48-linked ubiquitin chain differs strongly from that of the K63-linked ubiquitin chain, which may be the molecular basis for the two kinds of ubiquitin chains playing different signaling roles [32]. AKT ubiquitination occurs via K63 but not K48 [33]. As a direct E3 of AKT, TRAF6 participates in the process of AKT ubiquitination, membrane recruitment and phosphorylation after growth factor stimulation and increases the localization of AKT at the membrane, which are important steps in the activation of oncogenic AKT [33] (Fig. 3). Shi et al., studied the activation and ubiquitination of AKT in high TRAF6 expression cells such as oral cancer cell HN12 and breast cancer cell MDA-M-231 and low TRAF6 expression cells including oral cancer cell SCC9 and breast cancer cell MCF-7, their findings showed high ubiquitination and activation of AKT were observed in cells with highly expression of TRAF6, compared with low TRAF6 expression cells. Moreover, they also proved that TRAF6-mediated AKT ubiquitination and phosphorylation contributes to oral cancer and breast cancer malignant phenotype in vitro and in vivo [34]. In addition, evidences exhibited that TRAF6 is required for transforming growth factor-β (TGF-β) induced prostate cancer cell migration through PI3K-AKT signaling pathway [35].

### TRAF6 and NF-κB pathway

The transcription factor NF-κB was discovered in 1986 as a nuclear factor that binds to the enhancer of the immunoglobulin kappa light chain of activated B cells [36]. In a series of subsequent studies, it was found that NF-κB regulates a variety of biological processes, including immune and inflammatory responses, cell proliferation and apoptosis, and protumorigenic effects [23]. NF-κB signaling is well known to be abnormally activated in many tumors and constitutive activation of NF-κB benefits DNA replication and G1/S phase transition through up-regulation of Cyclin D1 expression or growth factors, leading to stimulate the proliferation of precancerous cells and induce Bcl-XL, which prevents apoptotic elimination of premalignant cells and facilitates the production of inflammatory factors, therefore, provides favorable microenvironment for the survival of tumor cells [37, 38]. In this way, NF-κB promotes the occurrence, development and metastasis of tumors through antiapoptotic effects, epithelial-mesenchymal transition (EMT), and tumor angiogenesis [39, 40]. TRAF6 indirectly enhances NF-κB activation by altering ROS levels [26, 41] (Fig. 3). Therefore, TRAF6 has become a potential therapeutic target for multiple tumors, such as multiple myeloma, liver cancer, and melanoma [42–44]. The adhesion of multiple myeloma cells to bone marrow stromal cells leads to activation of tumor-promoting signaling pathways, while TRAF6 promotes adhesion through NF-κB-induced adhesion factors [45]. Although previous study showed that TRAF6 does not affect NF-κB pathway in cancer cells under normal condition [46], Zhu et al. found that TRAF6 promotes colorectal cancer cells (SW48 and HCT116) proliferation and migration through NF-κB nuclear translocation [47].

### TRAF6 activates the AP-1 signaling pathway

AP-1 is composed of c-jun and c-fos protein family members and is an important transcription factor that binds to bZIP-like DNA-binding proteins in cells. The c-jun and c-fos protein family members interact to form various forms of homodimeric or heterodimeric AP-1. C-Jun is a transcriptional regulator and a member of the leucine zipper family, which can bind to the promoters of many genes and participate in the regulation of gene transcription. *C*-*Jun* is considered an oncogene because of its carcinogenic function. TRAF6 also induces JNK and p38 activation in a TAK1-dependent manner, leading to the activation of the AP-1 family members c-jun and c-fos to promote the production of inflammatory factors [48] (Fig. 3). TRAF6 promotes the progression of colorectal cancer both in vitro and in vivo through the AP-1 signaling pathway [47]. Similarly, in skin squamous cell carcinoma, silencing TRAF6 expression also significantly reduces AP-1 activity [16].

### TRAF6 and Wnt pathway

Activation of the Wnt pathway plays an extremely important role in cell proliferation, polarity, and apoptosis, which has been considered to be a key regulatory pathway in tumorigenesis [49]. Most importantly, the components of the Wnt pathway are involved in EMT and cell adhesion, stem cell regeneration, and cell proliferation and apoptosis, and abnormalities in these molecules often lead to the development of many types of cancer [50–52]. As a key transfer adaptor protein that transduces the Wnt3a/β-Catenin pathway, TRAF6 upregulated the mRNA expression of β-Catenin and subsequently activated Wnt target genes such as *c*-*Myc* and *LRP5* in human prostate cancer PC3U cells [53] (Fig. 3). Moreover, when TRAF6 was silenced by siRNA, Wnt3a-induced invasion was significantly reduced in PC3U and human colorectal SW480 cells [53].

### TRAF6 regulates MMP9 expression

MMP9 belongs to the matrix metalloproteinase (MMP) family and is a type IV collagenase. High MMP9 expression is related to invasion, metastasis and angiogenesis in diverse cancers [54, 55]. Moreover, MMP9 mediates the tumor microenvironment by promoting the extravasation of tumor cells [56]. Therefore, MMP9 has become a therapeutic target for various cancers, such as ovarian cancer, cervical cancer, and pancreatic cancer [57]. TRAF6 promotes the invasion and metastasis of melanoma, glioblastoma, gastric cancer and other tumors by overexpressing MMP-9, leading to malignant transformation of tumors [14, 58, 59] (Fig. 3) and down-regulation of TRAF6 in A549 lung adenocarcinoma cells effectively reduces MMP9 expression [60].

### TRAF6 and EMT

EMT is a biological process by which epithelial cells transform and acquire an aggressive mesenchymal phenotype. Many studies suggest that the acquisition of mesenchymal characteristics in tumor cells enhances tumor invasion. EMT has critical roles in metastasis and invasion of many types of cancers, including breast cancer, prostate cancer and head and neck squamous cell carcinoma (HNSCC) [61–63]. The study showed that EMT procedure was significantly blocked after inhibition of TRAF6 expression in HNSCC cell lines [63] (Fig. 3), while over-expression of TRAF6 up-regulated SLUG expression benefiting EMT in prostate cancer cells [62].

### TRAF6 and YAP pathway

Yes-associated protein (YAP) is one of the key downstream factors of the Hippo signaling pathway and is not only vital in the dynamic balance and development of tissue but also promotes tumorigenesis and participates in tumor development and metastasis [64]. Some studies have shown that YAP is involved in the biological behavior of pancreatic ductal adenocarcinoma, breast cancer, and rectal cancer [65–67]. TRAF6 promotes the malignant phenotype of pancreatic cancer by regulating YAP signaling [68] (Fig. 3). In addition, TRAF6 also plays a carcinogenic role through upregulating the expression of hypoxia-inducible factor-1α (HIF-1α) in colon adenocarcinoma and cervical carcinoma [69] (Fig. 3). The level of HIF-1α activity is related to tumorigenicity and angiogenesis [70]. Interestingly, however, data from Bruneau et al. indicate a controversial result in which TRAF6 inhibits proangiogenic signals in endothelial cells [71].

## The mechanism for regulation of TRAF6

### The regulation of TRAF6 by expression

**Fig. 4:**
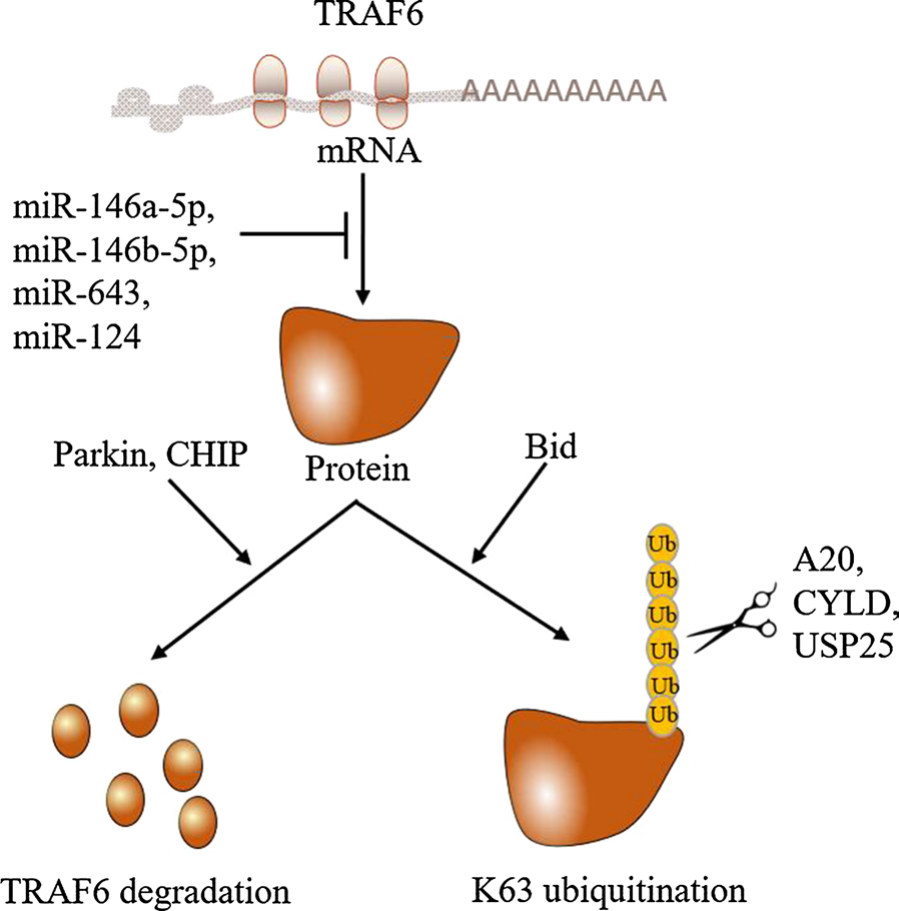
The mechanism for regulation of TRAF6. At present, the expression of TRAF6 is mainly affected by the E3 ubiquitin ligase (Parkin and CHIP) and miRNAs (miR-146a-5p, miR-146b-5p, miR-643 and miR–124). The ubiquitin ligase activity of TRAF6 is mainly affected by deubiquitinating enzymes (A20, CYLD and USP25) and Bid

Recent studies have found that the E3 ubiquitin ligase Parkin and Hsp70-interacting protein (CHIP) regulate TRAF6 by directly degrading it [72, 73] (Fig. 4). Zhang et al. found that the translocation of parkin downregulated the expression level of TRAF6; in contrast, inhibition of parkin upregulated the expression level of TRAF6 [72]. Li et al. found that the expression of TRAF6 was significantly reduced in 293T cells with high expression of CHIP, and the use of the proteasome inhibitor MG132 partially rescued the level of TRAF6 protein [73]. Thus, it can be concluded that CHIP promotes the degradation of TRAF6 in a proteasome-dependent manner [73]. They also found that TRAF6 ubiquitination was reduced in Chip−/− cells, thereby suggesting that CHIP promotes TRAF6 ubiquitination [73].

In addition, microRNAs (miRNAs) could decrease TRAF6 at the post-transcriptional level by binding to its 3′-untranslated region (3′-UTR) [74–77] (Fig. 4). In vitro and in vivo experiments have shown that miR-146a-5p deter the tumorigenesis of pancreatic ductal adenocarcinoma (PDAC) by repressing TRAF6 expression [74]. Moreover, Liu et al. demonstrated that miR-146b-5p exerts antitumor effects in gliomas by targeting TRAF6 directly [76]. Evidences also confirmed that miR-146b-5p is tumor suppressor by inhibiting the TRAF6/p-Akt signaling pathway in vitro and in vivo [77]. miR-643 has recently been proved to targeting TRAF6 in human endometrial epithelial cells (HEECs) that significantly reduces the expression of TRAF6 [78]. miR-124 has also been reported to be related to negative regulation of TRAF6 expression in human osteosarcoma cells and colorectal cancer cells [79, 80].

### The regulation of TRAF6 by its ubiquitin ligase activity

Ubiquitination is a reversible process, similar to other posttranslational modifications. To date, three major deubiquitinating enzymes have been found: A20, Cylindromatosis (CYLD) and USP25 (Fig. 4). A20 regulates polyubiquitination via its dual roles. In addition to inhibiting the ubiquitination of the TRAF6 complex by triggering the classical Lys48 polyubiquitination-mediated degradation of ubc13 [81], it also terminates the signal transduction process of TLR/IL-1R by removing the Lys63 ubiquitin chain of the TRAF6 catalytic linkage [82]. However, a deubiquitinase loss-of-function mutation of A20 (C103A) does not affect ubiquitination and K63-linked ubiquitination levels in TRAF6 [83]. Another deubiquitinase, CYLD, is recruited to TRAF6 by the adaptor protein p62, which inhibits TRAF6 ubiquitination, thereby inhibiting RANK signaling [84]. Therefore, CYLD is considered a tumor suppressor, and its dysfunction leads to excessive activation of TRAF6, resulting in a series of pathological responses. The ubiquitin-specific protease USP25 negatively regulates IL-17-mediated signaling and is thought to be a new deubiquitinase due to its ability to induce the removal of Lys63-linked ubiquitination in TRAF6 [85].

In contrast, BH3-interacting domain death agonist (Bid) is a member of the proapoptotic BH3-only Bcl-2 family and Kinsella et al. demonstrated that microglial Bid positively regulates TRAF6 K63-linked polyubiquitination by interacting with TRAF6 and thereby promotes TLR4-NF-κB signaling promoting TRAF6 ubiquitinase activity. [86] (Fig. 4).

## Targeting TRAF6 is a novel strategy for anti-tumor treatment

**Fig. 5:**
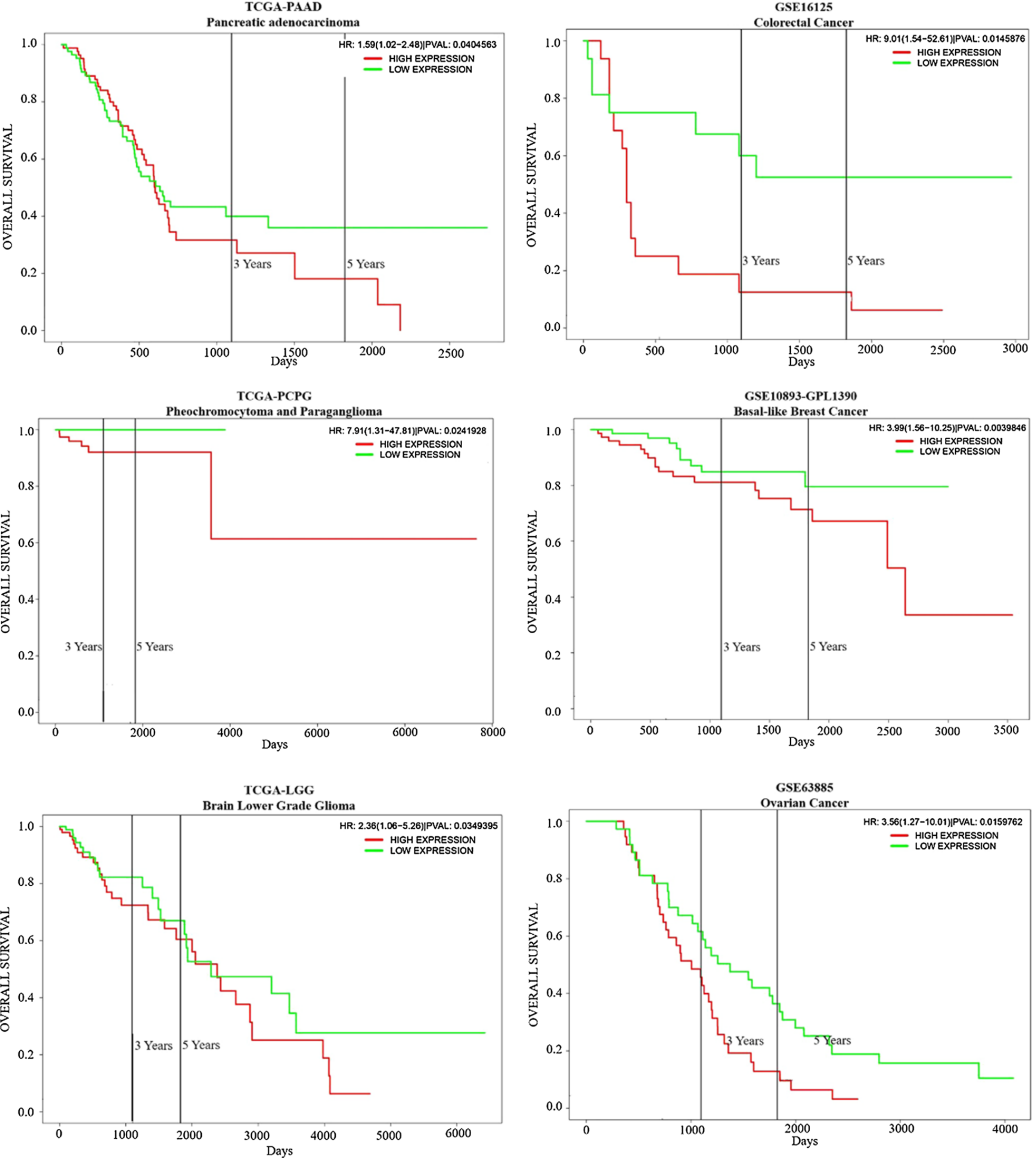
The overall survival of patients with cancers of low or high TRAF6 expression. The high expression of TRAF6 is significant related to poor prognosis comparing with the low expression of TRAF6. Data is from proggene V2

TRAF6 overexpression is closely related to tumorigenesis and tumor development. TRAF6 affects different signaling pathways involved in cancer and regulates tumor cell proliferation, survival, apoptosis, and invasion (Fig. 3). According to statistics from the TCGA and GEO, the high expression of TRAF6 is significant related to poor prognosis comparing with the low expression of TRAF6 (Fig. 5). Therefore, TRAF6 has become the target of targeted tumor therapy, and TRAF6 suppression treatment will provide a new therapeutic strategy for tumor therapy.

### Blocking TRAF6 activity through functional domain

#### Inhibition of the N-terminal functional domain of TRAF6

The first zinc finger and the complete RING domain are necessary for TRAF6 autoubiquitination and its interaction with the E2 ubiquitin conjugate enzyme Ubc13, while the binding of TRAF6 and Ubc13 generates Lys63-linked ubiquitin chains, which is an important step for TRAF6 to function [19]. Therefore, the RING finger and the first zinc finger of the N-terminus of TRAF6 protein may be key sites for TRAF6-targeted therapy. The RING domains of TRAF6 function as dimers and directly interact with Ubc13 [87]. The ubiquitination of AKT is a prerequisite for cancerous AKT activation and is mediated by TRAF6. TRAF6-mediated ubiquitination requires its ubiquitin ligase activity, while E3 ligase-inactivated TRAF6 (C70A) cannot mediate this process. The C70 residues are located at the binding site of TRAF6-Ubc13, which is in the RING domain [33]. However, the RING domain also plays a pivotal role in the immune function of TRAF6. Studies have shown that the body weight and immune system evolution of heterozygous Ubc13 ± mice are no different from those of wild-type littermates [88]. That is, single-dose Ubc13 mice still have normal immune function. Peroxidase-1 (PRDX1) is a member of the peroxidase protein family and inhibits its ubiquitin ligase activity by interacting with the RING domain of TRAF6 to negatively regulate the migration and invasion of cancer cells without destroying the structure of the RING domain [89]. Therefore, it may be a feasible strategy to destroy the ubiquitinase activity of TRAF6 without destroying the RING domain. The first zinc finger is considered a target for inhibitors because it regulates the polyubiquitination of TRAF6 [19]. Interestingly, Chen et al. do not find out inhibitory effects on growth and proliferation in multiple myeloma cells transfected with siTRAF6Zn-finger siRNA constructs targeting zinc fingers of the *TRAF6* gene [90].

#### Inhibition of the C-terminal functional domain of TRAF6

The TRAF6-C domain participates in the formation of homotrimers and mediates the interaction of TRAF6 with upstream signaling molecules. TRAF6 maintains its immune function mainly by binding to CD40 and latent infection integral membrane protein 1(LMP1) through different sites of its TRAF C domain [91]. TRAF6 is reported to play an essential role in CD40 and LMP1-mediated signaling [92]. However, it has been reported that CD40-mediated JNK activation and NF-kB activation are unaffected by blocking TRAF6 binding to CD40 in B cells [93]. Similarly, subsequent studies confirmed that the expression of mutant TRAF6 lacking TRAF-C (the domain required for CD40 binding) in TRAF6-deficient B cells restored CD40-mediated JNK activation and CD80 upregulation, which indicates that there may be an indirect mechanism by which TRAF6 is recruited to the CD40 signaling pathway [94]. In opposite to CD40, LMP1-mediated MAPK and NF-κB1 activation requires the TRAF6 TRAF-C domain [92]. Therefore, inhibition of the TRAF-C domain leads to a partial decrease in the immune function of TRAF6 but not complete loss. Research by Chen et al. found that siRNA targeting the TRAF6 C-terminus (siTRAF6C) downregulates TRAF6 protein expression, significantly inhibits myeloma proliferation and promotes apoptosis in vitro in a similar dose-dependent manner [90]. Hence, the C-terminus of TRAF6 is a promising target for tumor therapy, which can not only retain the majority of immune function but also effectively suppress the growth of cancer cells.

### Targeting TRAF6 antagonist research

Targeting TRAF6 by inhibitors have been extensively studied and can be grouped into the following four categories.

The first category includes protease inhibitors, including bortezomib and MG132 [95, 96]. Bortezomib (also known as PS341) is a proteasome inhibitor which downregulates the expression of TRAF6 to inhibit the maturation and function of osteoclasts in patients with multiple myeloma, thus becoming a potential treatment candidate for myeloma osteopathy [95]. MG132 is a potent and reversible aldol peptide 26S proteasome inhibitor that is capable of inhibiting TRAF6 expression in a dose-dependent manner. Studies show that MG132 can be used in combination with radiation therapy to treat pancreatic cancer by downregulating TRAF6 and inducing autophagy [96].

The second category is TRAF6 inhibitory peptides. TRAF-6 inhibitory peptides (Novus Biologics) target TRAF6 by binding to the T6DP motif of RANK, preventing RANK from binding to TRAF6. A meta-analysis by Shang et al. showed that Treg infiltration in tumors, especially in solid tumors, was negatively correlated with patient prognosis [97]. Wu et al. reported a study of the use of TRAF-6 inhibitor peptides (Novus Biologics) in liver cancer in 2019, in which the TRAF-6 inhibitor peptide (Novus Biologics) reduced the number of Tregs in the tumor by preventing the migration of Tregs to the tumor and therefore inhibited tumors in C57BL/6 mice with immune function [98].

The third category includes small molecule inhibitors targeting TRAF6, such as C25-140, epigallocatechin-3-gallate (EGCG) and resveratrol. The compound C25-140 reduces the activity of TRAF6-Ubc13 in vitro and in cells, thereby inhibiting the production of Lys63-linked ubiquitin chains [99]. EGCG is a novel E3 ubiquitin ligase inhibitor that prevents and treats melanoma by targeting TRAF6 [44]. Resveratrol may mediate the degradation of TRAF6, thereby inhibiting the effects of EMT and decreasing the proliferation and migration of prostate cancer cells [62]. Cinchona alkaloid, a chemical substance, promotes apoptosis of cancer cells in vitro and in vivo through competitive binding with the RING domain of TRAF6 [100].

The last category is Chinese herbal extracts, including shikonin and Nodakenin. Shikonin is a bioactive constituent of traditional Chinese herbs. Chen et al. found that shikonin can prevent the interaction of TRAF6 and RANK [101]. Nodakenin, a coumarin isolated from the roots of *Angelica gigas*, prevents NF-κB activation by interfering with the activation of TRAF6 in macrophages [102]. However, these herbal extracts have not been studied in cancer, which requires further study in vitro and in vivo.

## Conclusion

Based on the possession of a HECT domain or a RING domain, E3s are usually divided into two categories. E3s with the HECT domain possess an essential catalytic Cys residue and transfer Ub to the substrate by an intermediate Ub that contains a linked thioester. E3s with the RING domain interact with E2s, which makes E2s close to the substrate for ubiquitination. However, E3s do not exert catalytic activity [103, 104]. Compared with RING E3s, HECT E3s have intrinsic catalytic activity and therefore are more tractable drug targets [105]. Hence, E3s inhibitors are mostly developed to target HECT E3s [106]. However, the ring domain-containing E3s also play a carcinogenic role. To date, there are also some specific RING E3 inhibitors (e.g., IAP and MDM2) that have entered clinical research and provide a promising strategy for cancer chemotherapy and prevention [107–109].

As a RING domain-containing E3, TRAF6 affects different signaling pathways involved in cancer, and high expression of TRAF6 leads to poor prognosis of various tumors. TRAF6 targeted therapy is a promising treatment strategy. However, in addition to its role in tumors, TRAF6 is involved in many biological behaviors. For example, TRAF6 is involved in lymph node organogenesis and the development of hair follicles, sweat and sebaceous glands [110]. It also participates in the transduction of B cell CD40 signaling and LMP1 signaling [92]. In addition, the maturation and activation of dendritic cells and the regulation of T cell functions also require TRAF6 [111, 112]. Earlier studies have shown that TRAF6−/− mice develop low-sweating ectodermal dysplasia, severe osteoporosis, and early age death [110, 113]. Therefore, complete inhibition of TRAF6 causes serious side effects and is not a preferred option for treating tumors.

For future TRAF6-targeted therapy, how to suppress tumors by inhibiting TRAF6 without affecting the role of TRAF6 in immunity is worth studying. In the “Strategy for TRAF6” section, we proposed a feasible strategy for TRAF6-targeted therapy, which is destroying the ubiquitinase activity of TRAF6 without destroying the RING domain. To achieve this goal, there are two possible ways: one is to reduce the amount of Ubc13, and the other is to develop protein–protein interaction (PPI) inhibitors. At present, the small molecule Ubc13 inhibitors NSC697923 and ML307 have been developed, which may provide new ideas for inhibiting the carcinogenic function of TRAF6 in tumors [114, 115]. However, to date, whether Ubc13 inhibitors can inhibit tumors by reducing the ubiquitination activity of TRAF6 has not been studied, which is worthy of further research. A recent study indicates that deamidation creates a new intramolecular salt bridge in Ubc13 that competes with a critical intermolecular salt bridge at the native Ubc13/TRAF6RING interface. In this way, deamidation inhibits the binding of the TRAF6 RING domain and Ubc13 [116]. Therefore, deamidases may provide a direction for targeted therapy of TRAF6 in tumors. Furthermore, RING Finger protein 11 (RNF11) and the small molecule inhibitor C25-140 downregulate the ubiquitinase activity of TRAF6 by reducing the activity of TRAF6-Ubc13 [99, 117].

In addition to inhibiting the ubiquitination activity of TRAF6, inhibiting its C-terminus may also be a feasible strategy. TRAF6 can increase ROS levels through its interaction with ECSIT and indirectly activate NF-κB signaling. Therefore, the use of PPI inhibitors to inhibit the combination of TRAF6 and ECSIT may play a role in tumor suppression. Prdx6 competitively interacted with ECSIT to TRAF6 through its C-terminal TRAF-C domain, leading to the interruption of the TRAF6-ECSIT interaction [118]. Overall, PPI inhibitors may have potential prospects in future TRAF6 targeted therapy for tumors and require follow-up research and development.

## Data Availability

Available.

## Abbreviations

TNFR: Tumor necrosis factor receptor
TRAFs: Tumor necrosis factor receptor-related factors
TNFSF: Tumor necrosis factor superfamily
TLR/ILR: Toll-like/interleukin-1 receptor
TRAF6: Tumor necrosis factor receptor-associated factor 6
MAPK: Mitogen-activated protein kinase
E3: E3 ubiquitin ligase
E1: Ubiquitin activating enzyme
E2: Ubiquitin conjugating enzyme
TLR4: Toll-like receptor 4
IRAK: Interleukin-1 receptor-associated kinase
NF-κB: Nuclear factor kappa-B
AP-1: Activator protein-1
PI3K: Phosphatidylinositol 3-kinase
Bad: Bcl-xL/Bcl-2 associated death promoter
ECSIT: Evolutionarily conserved signaling intermediate in Toll pathways
ROS: Reactive oxygen species
pAKT: Phosphorylated AKT
TGF-β: Transforming growth factor-β
EMT: Epithelial-mesenchymal transition
MMP: Matrix metalloproteinase
HNSCC: Head and neck squamous cell carcinoma
YAP: Yes associated protein
HIF-1α: Hypoxia-inducible factor-1α
CHIP: Hsp70-interacting protein
miRNAs: MicroRNAs
3′-UTR: The 3′-untranslated region
PDAC: Pancreatic ductal adenocarcinoma
HEECs: Human endometrial epithelial cells
CYLD: Cylindromatosis
Bid: BH3-interacting domain death agonist
PRDX1: Peroxidase-1
LMP1: Latent infection integral membrane protein 1
siTRAF6C: siRNA targeting the TRAF6 C-terminus
EGCG: Epigallocatechin-3-gallate
PPI: Protein–protein interaction
